# Supplementary material for: Modelling Skylarks (Alauda arvensis) to Predict Impacts of Changes in Land Management and Policy: Development and Testing of an Agent-Based Model
Source: PLoS One. 2013 Jun 6;8(6):e65803. doi: 10.1371/journal.pone.0065803 (PMC3675089; doi:10.1371/journal.pone.0065803)
Supplement: Supporting Information S4 — The skylark ODdox as a zipped archive. (ZIP) [file pone.0065803.s004.zip › Skylark_ODdox/functions_func_0x6d.html]

ALMaSS Skylark ODdox: Class Members - Functions


ALMaSS Skylark ODdox
 2.0


- Main Page
- Related Pages
- Classes
- Files

- Class List
- Class Index
- Class Hierarchy
- Class Members

- All
- Functions
- Variables
- Typedefs

- a
- b
- c
- d
- e
- f
- g
- h
- i
- j
- k
- l
- m
- n
- o
- p
- r
- s
- t
- u
- v
- w
- y
- z
- ~

### - m -

- MagicMapP2PolyRef()
  : Landscape
- MainMapDecay()
  : Pesticide
- MakeBugPercentArray()
  : CropData
- MakeCluster()
  : Landscape
- MakeStockFarmer()
  : PesticideTrialControl
  , PesticideTrialToxicControl
  , Farm
  , PesticideTrialTreatment
  , ConventionalPlant
  , OrganicPlant
- Management()
  : Farm
- Manipulation1()
  : RasterMap
- MapErrorMsg()
  : MapErrorMsg
- MapHeight()
  : RasterMap
- MapWidth()
  : RasterMap
- MarchFirst()
  : Calendar
- Marsh()
  : Marsh
- MixedForest()
  : MixedForest
- ModifyPolyRef()
  : Landscape
- Molluscicide()
  : Farm
- MovementMap()
  : MovementMap
- MovementMap16()
  : MovementMap16


- Generated on Thu Jan 10 2013 13:15:39 for ALMaSS Skylark ODdox by
   1.8.1.1
